# Design, Synthesis and Antiviral Potential of 14-Aryl/Heteroaryl-14*H*-dibenzo*[a,j]*xanthenes Using an Efficient Polymer-Supported Catalyst

**DOI:** 10.3390/molecules17067543

**Published:** 2012-06-18

**Authors:** Kalla Reddi Mohan Naidu, Balam Satheesh Krishna, Mungara Anil Kumar, Palanisamy Arulselvan, Shaik Ibrahim Khalivulla, Ola Lasekan

**Affiliations:** 1 Department of Food Technology, Universiti Putra Malaysia, 43400 Serdang, Selangor, Malaysia; 2 Department of Chemistry, Sri Venkateswara University, Tirupati 517502, India; 3 Department of Nanomaterial Chemistry, Dongguk University, Gyeongju 780-714, Korea; 4 Laboratory of Vaccines and Immunotherapeutics, Institute of Bioscience, Universiti Putra Malaysia, 43400 UPM Serdang, Selangor, Malaysia; 5 Department of Pharmaceutical Chemistry, Faculty of Pharmaceutical Sciences, UCSI University, No.1, Jalan Menera Gading, 56000 Cheras, Kuala Lumpur, Malaysia

**Keywords:** aryl aldehydes, 2-naphthol, PEG-OSO_3_H, neat reaction, xanthenes, tobacco mosaic virus

## Abstract

Polyethyleneglycol bound sulfonic acid (PEG-OSO_3_H), a chlorosulphonic acid-modified polyethylene glycol was successfully used as an efficient and eco-friendly polymeric catalyst in the synthesis of 14-aryl/heteroaryl-14*H*-dibenzo[*a,j*]xanthenes obtained from the reaction of 2-naphthol and carbonyl compounds under solvent-free conditions with short reaction times and excellent yields. The biological properties of these synthesized title compounds revealed that compounds **3b**, **3c**, **3f** and **3i** showed highly significant anti-viral activity against tobacco mosaic virus.

## 1. Introduction

Viral plant diseases originated from tobacco mosaic virus (TMV) are considered serious threats to modern agriculture worldwide. TMV is known to infect nine plant families and at least 125 individual species, such as tobacco, tomato, pepper, cucumbers and a number of ornamental flowers. The potential yield losses can fluctuate from 5 to 90%, depending on the strain/severity of TMV such as the total time of infection, the optimal temperature during disease development/progression and the presence of other plant diseases. It is found that in certain fields 90−100% of the plants show mosaic or leaf necrosis by harvesting time. Several investigations have shown that TMV can modify plant phenotypes by destruction of mitochondria followed by damage of plant quality. As a result of this sequence, plant virus is also known as “plant cancer” and is difficult to prevent. So far, there are neither effective chemical treatments that fully protect plants from TMV infection nor any other factor that eliminates TMV from infected plants under field conditions [1].

In recent years polymer-supported compounds used as supported catalysts, reagents and scavengers have drawn the attention of researchers [2]. The utility of polymer-supported catalysts is well-recognized with their advantages like ease of workup, simple separation of products from the catalysts, economy and easily use in industrial processes [3]. The high reactivity, lack of diffusion phenomena, analytical simplicity and solubility profile of polyethylene glycols (PEGs with MW > 2,000 Da) as mostly soluble in polar solvents and insoluble in a few non-polar solvents have established them as good catalysts in homogeneous solid supported catalysis [4]. Rather than catalyst they are also used as a solvent for high temperature reactions as they melt at low temperatures [5]. Among them PEG-OSO_3_H is a good example of solid supported organic acid catalyst that is functionalized with mild acidity, non-volatility and non-corrosiveness and also recognized as a good surfactant. It is reported that PEG-OSO_3_H had been used for the synthesis of 3,4-dihydropyrimidones via the Biginelli reaction [5], regioselective ring opening of epoxides with thiocyanate anion [6], Beckmann rearrangement and dehydration of oximes [7] and synthesis of bis(indolyl)methanes and 4,4′-(arylmethylene)-bis(3-methyl-1-phenyl-1*H*-pyrazol-5-ols) [8].

Aryl-14*H*-dibenzo[*a,j*]xanthenes are reported as important antibacterial, antiviral and anti-inflammatory agents [9,10,11]. In addition to their biological applications they also applied in photodynamic therapy [12], in industries as dyes, in laser technology and as fluorescent materials for visualization of biomolecules [13,14,15]. These benzoxanthenes were synthesized from the reaction of 2-naphthol with formamide [16], β-hydroxynaphthyl carbinol with resorcinol [17], and from the action of hot alkali on 2-naphthyl oxide [18]. The xanthene synthesis is catalyzed by some catalysts like *p*-toluenesulfonic acid [19], sulfamic acid [20], AcOH/H_2_SO_4_ [15], iodine [21,22], K_5_CoW_12_O_40_·3H_2_O [23], cyanuric chloride [24], LiBr [25], HClO_4_·SiO_2_ [26], KAl(SO_4_)_2_·12H_2_O [27], silica sulfuric acid [28] and Yb(OTf)_3_ [29]. However, there is a need to find new catalysts and green methods to minimize the drawbacks of existing methods such as poor yields, prolonged reaction times, toxic organic solvents, excess reagents, catalysts and harsh reaction conditions so here we are reporting PEG-OSO_3_H as a catalyst for this reaction and to the best of our knowledge, this is also the first investigation on the anti-TMV bioactivity of xanthenes designed in this article.

## 2. Results and Discussion

### 2.1. Chemistry

**Scheme 1:**
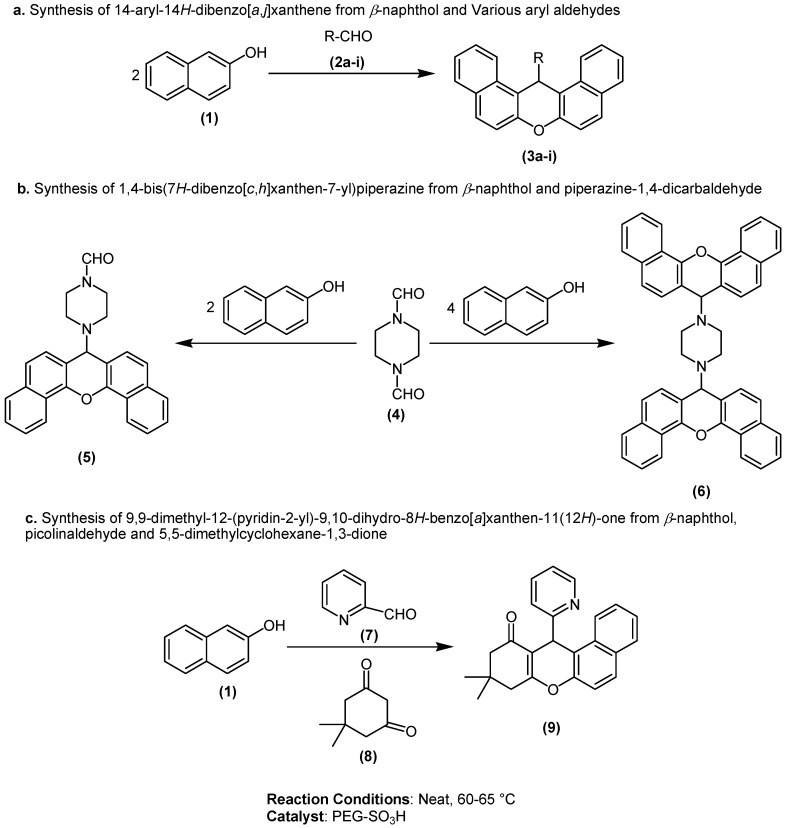
Synthesis of 14-aryl/heteroaryl-14*H*-dibenzo[*a,j*]xanthenes.

To examine non-hazardous methods for the transformations which occur in organic synthesis herein we report a highly versatile and efficient synthesis of aryl/heteroaryl-14*H*-dibenzo[*a,j*]xanthenes **3a**–**i** (Scheme 1) from two equivalents of 2-naphthol (**1**) and one equivalent of various aryl aldehydes **2a**–**i**, and catalytic amounts of a polymer-supported acidic catalyst. In a typical reaction, a 2:1 mixture of **1** and **2a** and a catalytic amount of PEG-OSO_3_H (1 mol %) was placed in a 50 mL flat-bottomed flask and heated for 30–60 min on a hot plate at 60–65 °C under solvent free condition to produce **3a** in excellent yield. The same reactions conditions were applied for the synthesis of the remaining series of compounds **2b**–**i** as presented in Scheme 1 and summarized in Table 1. 

During our study on the dehydration of 2-naphthol by various aldehydes under solvent-free condition at 80 °C, we made an interesting observation that 5 mL of PEG-600 catalyzed the reaction. 2-Naphthol underwent dehydration with benzaldehyde leading to aryl or heteroaryl-14*H*-dibenzo [*a,j*]xanthenes after long reaction times and the yields were very low.

The same method was employed in the presence of catalytic amount of chlorosulphonic acid supported PEG-6000 (1 mol %) and we found it as an efficient catalyst which brought this reaction to completion at 65 °C in the absence of solvent. Blank experiments have shown that the chlorosulphonoic acid alone could not bring about this transformation. In addition, it was observed that only 1 mol % of PEG-OSO_3_H is sufficient for the formation of xanthenes. Encouraged by the results obtained for benzaldehyde and other aldehydes, the reaction was further studied under the current catalytic conditions with a variety of carbonyl (aromatic and heterocyclic aldehydes) compounds. All the carbonyl substrates reacted smoothly with 2-naphthol to produce xanthenes (Table 1) in high yields under these reaction conditions (60–65 °C; 30–60 min). The electronic nature of the substituent in the aromatic ring did not show any noticeable effect on this conversion.

Additionally, we also investigated the recyclability of PEG–OSO_3_H for three consecutive cycles (fresh + 3 cycles) for the synthesis of 14-phenyl-14*H*-dibenzo[*a,j*]xanthenes (**3a**). 2-Naphthol was treated with benzaldehyde in the presence of 1 mol % catalyst under neat conditions for 20 min. After completion of the reaction, PEG–OSO_3_H was extracted with water and reused after the evaporation of aqueous layer under reduced pressure. The above sequence was repeated three times to produce the product **3a** in good yields without any significant loss in the catalytic activity of PEG–OSO_3_H (Table 2). 

The same reaction procedure was followed for the synthesis of remaining xanthene derivatives, *i.e.*, monofunctionalized xanthene derivative 4-(14*H*-dibenzo[*a,j*]xanthen-14-yl)piperazine-1-carbaldehyde (**5**) and bifunctionalized xanthene derivative 7-(1-(14*H*-dibenzo[*a,j*]xanthen-14-yl)piperidin-4-yl)-7*H*-dibenzo[*c,h*]phenoxazine (**6**) from the reaction of piperazine-1,4-dicarbaldehyde with 2-naphthol. Similarly 9,9-dimethyl-12-(pyridin-2-yl)-9,10-dihydro-8*H*-benzo[*a*]xanthen-11(12H)-one (**9**) was also synthesized from the reaction of 2-naphthol, 2-picolinaldehyde and 5,5-dimethylcyclohexane-1,3-dione as given in Scheme 1. Preliminary data for these compounds were provided in Table 1. 

### 2.2. Biology 

**Figure 1:**
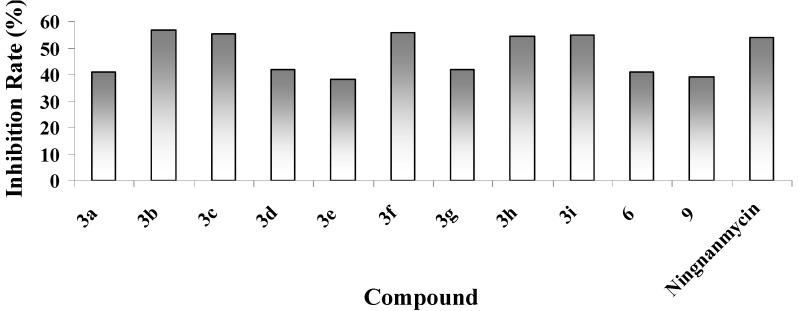
The anti-TMV activity of the synthesized compounds.

Ningnanmycin is a commercial antiviral agent, isolated from *Strepcomces noursei* var. *xichangensisn* by the Chengdu Institute of Biology, Chinese Academy of Sciences. It is a kind of well-known microbial pesticide that imparts its action by destruction of the coat protein of TMV, thereby inducing plant host resistance pathways. It is more effective in the treatment of plants against TMV than the other accessible commercial agents used in the agro-pharmaceutical industry [33]. However, the use of this agent for field trials is largely very limited by its photosensitivity and water stickiness [34]. Therefore, further extensive investigations needs to be conducted in this related area for the development of a highly efficient, economical, novel, and environmentally benign antiviral agent. In this present investigation, we used ningnanmycin as a positive control for comparison of viral activities. In this study it was observed that basic xanthene moiety of compounds showed highly significant anti-viral activity against tobacco mosaic virus replication, as depicted in Figure 1, most of our designed compounds exhibited good antiviral properties.

The basic polynuclear structural framework (tetracyclic and pentacyclic nuclei) in all synthesized compounds containing an alkenyl functionality in conjugation to the oxygen atom of the xanthene nucleus is the basic structural requirement for the exhibition of antiviral activity which was previously reported for the compounds with the basic skeletons like fluorenone and anthraquinone derivatives. Similarly the abovementioned structural features were also attributed the antiviral activity of the compounds in the present study. Further, on comparison of all these analogues, **3b** afforded potent viral activity which is comparable to that of ningnanmycin. However, in most cases, the piperazine-1,4-dicarbaldehyde had no distinct influence on the antiviral activity. In addition, the introduction of electron-withdrawing groups (**3b**, **3c**, **3f**, **3g** and **3i**) resulted in higher antiviral potential than the corresponding analogues with electron-donating groups (**3d**, **3e** and **3h**). The corresponding compound **6** showed lower inhibitory efficiency, which is possibly due, in part, to the relatively rigid structure. On the basis of these observed results it may be speculated that the antiviral activity of xanthenes group of compounds are primarily due to the presence of nitrogenous groups, which can prevent/manage the host plant from initial infection/ replication by virus.

## 3. Experimental

### 3.1. General

All chemicals were purchased from Sigma-Aldrich and were used without further purification. IR spectra were recorded on a Perkin-Elmer 683 spectrophotometer using KBr optics. ^1^H- and ^13^C-NMR spectra were recorded on Bruker Avance 300 MHz NMR spectrometer operating at 300 MHz for ^1^H-NMR and 75 MHz for ^13^C-NMR. NMR data recorded in CDCl_3_ and referenced to TMS (^1^H and ^13^C). Mass spectra were recorded on a JEOL GCMATE II GC-MS spectrometer at SAIF, IIT Madras (Chennai, India).

### 3.2. Catalyst Preparation

At 0 °C, chlorosulfonic acid (1.16 g, 10 mmol) was added to a solution of PEG-6000 (6.0 g, 1 mmol) in 5 mL of CH_2_Cl_2_. Then the resulting solution was stirred at room temperature overnight, and the solution was concentrated under vacuum. Then diethyl ether (5 mL) was added and the precipitated product was filtered and washed with diethyl ether three times to afford the purified PEG-OSO_3_H [7].

### 3.3. General Procedure for the Synthesis of 14-(4-Chloro-3-nitrophenyl)-14H-dibenxo[a,j]xanthenes ***3a**–**i***

2-Naphthol (**1**, 0.29 g, 2 mmol) and benzaldehyde (**2a**, 0.10 g, 1 mmol) were mixed in the presence of PEG-OSO_3_H catalyst. The resulting mixture was then stirred for 30 min at 60–65 °C. After the completion of the reaction (as indicated by TLC), the reaction mixture was poured into ice water and the obtained solid was collected by filtration, washed with water and then dried. The obtained solid was recrystallised from hot ethanol. 

The physical and spectral data of known compounds **3a**–**h** were found to be in agreement with the reported data [30,31,32] while the characterization data of newly synthesized products **3i**, **5**, **6** and **9** are given below.

After completion of the reaction, water was added to the reaction mixture and was shaken to dissolve PEG-OSO_3_H in it. Then in order to recover the catalyst, water was evaporated under reduced pressure, and the resulting solid was washed with diethyl ether, and dried. The recovered catalyst was reused four times for the subsequent reactions without loss of potentiality in catalytic activity [35].

*14-Phenyl-14H-dibenzo[a,j]xanthene* (**3a**). Pale yellow solid, m.p.: 181–182 °C. IR (KBr) *v*_max_: 3068, 3020, 2885, 1620, 1590, 1512, 1488, 1457, 1402, 1252, 1080, 1025, 965, 825, 745, 700 cm^−1^. ^1^H-NMR (CDCl_3_): *δ* 8.40 (d, *J* = 8.4 Hz, 2H), 7.82 (d, *J* = 7.9 Hz, 2H), 7.79 (d, *J* = 8.8 Hz, 2H), 7.58 (t, *J* = 7.7 Hz, 2H), 7.53 (d, *J* = 7.5 Hz, 2H), 7.49 (d, *J* = 8.8 Hz, 2H), 7.41–7.00 (m, 5H), 6.49 (s, 1H, Ar-CH). ^13^C-NMR (CDCl_3_): *δ* 148.1, 145.5, 131.4, 131.8, 128.7, 128.2, 128.1, 126.8, 126.5, 126.3, 124.2, 122.3, 117.1, 117.5, 38.3. EI-MS: *m/z* (%) 358 (M^+^); Anal. Calcd. for C_27_H_18_O: C, 90.47; H, 5.06. Found: C, 90.41; H, 5.14.

*14-(4-Nitrophenyl)-14H-dibenzo[a,j]xanthene* (**3b**). Yellow solid, m.p.: 310–312 °C. IR (KBr) *v*_max_: 3062, 2931, 1629, 1595, 1527, 1453, 1401, 1346, 1200, 1141, 1108, 1015, 965, 850, 828, 810, 743 cm^−1^. ^1^H-NMR (CDCl_3_): *δ* 8.30 (d, *J* = 8.5 Hz, 2H), 7.98 (d, *J* = 8.7 Hz, 2H), 7.85–7.45 (m, 12H, ArH), 6.60 (s, 1H, Ar-CH). ^13^C-NMR (CDCl_3_): *δ* 152.5, 148.0, 145.8, 135.1, 130.9, 130.7, 129.7, 128.6, 127.3, 124.7, 123.5, 123.0, 117.8, 116.2, 36.5. EI-MS: *m/z* (%) 403 (M^+^). Anal. Calcd. for C_27_H_17_NO3: C, 80.38; H, 4.25; N, 3.47. Found: C, 80.30; H, 4.35; N, 3.55.

*14-(2-Nitrophenyl)-14H-dibenzo[a,j]xanthene* (**3c**). Yellow solid, m.p.: 290–291 °C. IR (KBr) *v*_max_: 3402, 3056, 2928, 1615, 1594, 1524, 1352, 1243, 1140, 815, 750 cm^−1^. ^1^H-NMR (CDCl_3_): *δ* 8.59–7.15 (m, 16H), 7.54 (s, 1H). ^13^C-NMR (CDCl_3_): *δ* 150.1, 147.8, 141.3, 134.5, 132.7, 132.0, 130.5, 129.8, 129.6, 129.2, 128.1, 127.6, 125.5, 125.1, 124.5, 122.9, 118.5, 118.1, 33.1. EI-MS: *m/z* (%) 403 (M^+^). Anal. Calcd. for C_27_H_17_NO_3_: C, 80.38; H, 4.25; N, 3.47. Found: C, 80.23; H, 4.31; N, 3.54.

*14-(4-Methoxyphenyl)-14H-dibenzo[a,j]xanthene* (**3d**). Yellow solid, m.p.: 203–205 °C. IR (KBr) *v*_max_: 3041, 2913, 1615, 1582, 1511, 1465, 1433, 1404, 1250, 1122, 1088, 968, 825, 805, 748 cm^−1^. ^1^H-NMR (CDCl_3_): *δ* 8.41 (d, *J* = 8.3 Hz, 2H), 7.83 (d, *J* = 8.2 Hz, 2H), 7.72 (d, *J* = 8.3 Hz, 2H), 7.58–6.71 (m, 10H, ArH), 6.51 (s, 1H), 3.70 (s, 3H). ^13^C-NMR (CDCl_3_): *δ* 158.2, 149.5, 137.1, 134.2, 131.4, 130.1, 129.6, 129.2, 127.8, 124.9, 123.1, 118.6, 117.8, 114.5, 54.1, 37.4. EI-MS: *m/z* (%) 388 (M^+^). Anal. Calcd. for C_28_H_20_O_2_: C, 86.57; H, 5.19. Found: C, 86.44; H, 5.23.

*14-(3-Methoxyphenyl)-14H-dibenzo[a,j]xanthene* (**3e**). Yellow solid, m.p.: 163–164 °C. IR (KBr) *v*_max_: 3013, 2951, 2936, 1594, 1487, 1453, 1439, 1400, 1250 cm^−1^. ^1^H-NMR (CDCl_3_) *δ* 8.38 (d, 2H, *J =* 8.4 Hz). 7.80–7.72 (m, 4H), 7.65–7.31 (m, 6H), 7.01–6.53 (m, 4H), 6.41 (s, 1H), 3.61 (s, 3H). ^13^C-NMR (CDCl3): *δ* 152.6, 148.8, 146.4, 131.3, 131.0, 129.2, 128.8, 128.5, 126.7, 124.3, 122.8, 120.8, 118.4, 117.1, 115.2, 111.4, 55.6, 38.3, Anal.Calcd. for C_28_H_20_O_2_: C, 86.45; H, 5.06. Found: C, 86.53; H, 5.14.

*14-(4-Chlorophenyl)-14H-dibenzo[a,j]xanthene* (**3f**). Brown solid, m.p.: 286–287 °C. IR (KBr) *v*_max_: 3050, 2926, 1620, 1594, 1456, 1438, 1393, 1242, 1061, 960, 826, 778, 695 cm^−1^. ^1^H-NMR (CDCl_3_): *δ* 8.45 (d, *J* = 8.5 Hz, 2H), 8.01 (d, *J* = 8.7 Hz, 2H), 7.88–7.50 (m, 12H), 7.46 (s, 1H). ^13^C-NMR (CDCl_3_): *δ* 156.1, 147.5, 132.3, 131.2, 129.8, 128.1, 128.6, 127.0, 126.8, 126.5, 124.7, 119.3, 118.2, 117.8, 33.3. EI-MS: *m/z* (%) 392 (M^+^). Anal. Calcd. for C_27_H_17_ClO: C, 82.54; H, 4.36. Found: C, 82.42; H, 4.41.

*14-(3-Chlorophenyl)-14H-dibenzo[a,j]xanthene* (**3g**). Brown solid, m.p.: 173–174 °C. IR (KBr) *v*_max_: 3053, 2924, 1621, 1592, 1508, 1455, 1430, 1398, 1245, 1064, 959, 815, 775, 748, 691 cm^−1^. ^1^H-NMR (CDCl_3_): *δ* 8.30–6.96 (m, 16H, ArH), 6.45 (1H, s). ^13^C-NMR (CDCl_3_): *δ* 148.1, 146.3, 134.2, 131.6, 131.0, 129.6, 129.1, 128.1, 128.0, 127.2, 126.8, 126.4, 124.5, 122.4, 118.1, 116.4, 37.6. EI-MS: *m/z* (%) 392 (M^+^). Anal. Calcd. for C_27_H_17_ClO: C, 82.54; H, 4.36. Found: C, 82.47; H, 4.43.

*14-(4-Hydroxy-3-methoxyphenyl)-14H-dibenzo[a,j]xanthene* (**3h)**. Yellow solid, m.p.: 206–207 °C. IR (KBr) *v*_max_: 3020, 2399, 1593, 1508, 1431, 1215 cm^−1^. ^1^H-NMR (CDCl_3_): 8.39 (d, 2H, *J* *=* 8.30 Hz), 7.76–7.84 (m, 4H), 7.37–7.58 (m, 6H), 7.12–7.16 (m, 1H), 6.82 (d, 1H, *J =* 2.14 Hz), 6.71 (d, 1H, *J* = 8.0 Hz),3.64 (s, 3H), 6.42 (s, 1H), 5.32 (s, 1H). ^13^C-NMR (CDCl_3_): 151.9, 150.5, 147.5, 140.2, 134.7, 134.3, 132.0, 129.9, 127.5, 126.0, 124.2, 121.1, 120.8, 117.5, 114.6, 58.9, 40.8. Anal. Calcd. for C_28_H_20_O_3_: C, 83.15; H, 4.98. Found: C, 83.02; H, 4.88.

*14-(4-Chloro-3-nitrophenly)-14H-dibenxo[a,j]xanthene* (**3i**). Yellow solid, m.p.: 178–179 °C. IR (KBr) *v*_max_: 3157, 1412, 1231, 820, 746 cm^−1^. ^1^H-NMR (CDCl_3_): *δ* 8.24–7.24 (m, 15H, Ar-H), 6.54 (s, 1H, Ar-CH). ^13^C-NMR (CDCl_3_): *δ* 151.6, 147.2, 142.3, 132.3, 128.1, 127.1, 126.1, 125.4, 122.6, 122.1, 117.6, 114.3, 125.1, 134.2, 129.6, 124.2, 46.1. EI-MS: 476 (M+K).

### 3.4. Procedure for the Synthesis of 4-(14H-Dibenzo[a,j]xanthen-14-yl)piperazine-1-carbaldehyde *(**5**)*

2-Naphthol (**1**, 0.29 g, 2 mmol) and piperazine-1,4-dicarbaldehyde (**4**, 0.14 g, 1 mmol) were mixed in the presence of PEG-OSO_3_H catalyst (1 mol %). The resulting mixture was then stirred for 30 min at 60–65 °C. After the completion of the reaction (as indicated by TLC), the reaction mixture was poured into ice water and the obtained solid was collected by filtration, washed with water and then dried. Green solid, m.p.: 183–184 °C. IR (KBr) *ν*_max_: 3153, 1614, 1410, 1234, 746 cm^−1^. ^1^H-NMR (CDCl_3_): *δ* 7.83 (s, 1H, –CHO), 7.36–6.60 (m, 12H, Ar-H), 5.87 (s, 1H, Ar-CH), 1.35–1.20 (m, 8H, –N–CH_2_). ^13^C-NMR (CDCl_3_): d 153.4, 151.3, 136.6, 127.3, 123.4, 121.7, 120.0, 119.40, 119.0, 116.6, 111.8, 56.6, 55.5, 32.1. EI-MS: 394 (M^+^), 412 (M+NH_4_^+^).

### 3.5. Procedure for the Synthesis of 7-(1-(14H-dibenzo[a,j]xanthen-14-yl)piperidin-4-yl)-7H-dibenzo-[c,h]phenoxazine *(**6**)*

2-Naphthol (**1**, 0.58 g, 4 mmol) and piperazine-1,4-dicarbaldehyde (4, 0.14 g, 1 mmol) were mixed in presence of PEG-OSO_3_H catalyst (1 mole%). The resulting mixture was then stirred for 30 min at 60–65 °C. After the completion of the reaction (as indicated by TLC), the reaction mixture was poured into ice water and the obtained solid was collected by filtration, washed with water and then dried. Light greenish solid, m.p.: 185–187 °C; IR (KBr) *v*_max_: 3157, 1412, 1231, 820, 746 cm^−1^; ^1^H-NMR (CDCl3): *δ* 8.26–7.35 (m, 24H, Ar-H), 7.25 (s, 2H, Ar-CH), 1.40–1.6 (m, 8H, –N–CH_2_); APCI-MS: 664 (M+NH_4_^+^).

### 3.6. Procedure for the Synthesis of 9,9-Dimethyl-12-(pyridin-2-yl)-9,10-dihydro-8H-benzo[a]xanthen-11(12H)-one *(**9**)*

2-Naphthol (1, 0.14 g, 1 mmol), 2-picolinaldehyde (**7**, 0.10 g, 1 mmol) and 5,5-dimethylcyclo-hexane-1,3-dione (**8**, 0.14 g, 1 mmol) were mixed in presence of PEG-OSO_3_H catalyst (1 mol %). The resulting mixture was then stirred for 30 min at 60–65 °C. After the completion of the reaction (as indicated by TLC), the reaction mixture was poured into ice water and the obtained solid was collected by filtration, washed with water and then dried. White solid, m.p.: 179–181 °C. IR (KBr) *v*_max_: 2950, 1670, 1630, 1545, 1350, 1220, 1150, 102 cm^−1^. ^1^H-NMR (CDCl_3_): *δ* 6.55–8.05 (m, 10H, ArH), 5.62 (s, 1H, CH), 2.55 (s, 2H, CH_2_), 2.26 (s, 2H, CH_2_) 1.10 (s, 3H, CH_3_), 0.99 (s, 3H, CH_3_). ^13^C-NMR (CDCl_3_): *δ* 197.6, 164.1, 149.4, 148.2, 133.8, 132.1, 132.0, 129.5, 128.9, 128.8, 127.4, 125.2, 124.3, 118.8, 117.5, 115.2, 112.9, 51.5, 41.9, 41.1, 34.1, 32.8, 29.7, 27.9. EI-MS (%): 355 (M^+^).

### 3.7. Antiviral Activity

#### 3.7.1. Purification of Tobacco Mosaic Virus (TMV)

Using Goodings’ method [36], the upper leaves of *Nicotiana tabacum* L inoculated with TMV were selected and ground in a phosphate buffer, then filtered through double layer pledget. The filtrate was centrifuged at 10,000 g, treated twice with PEG and centrifuged again. The whole experiment was carried at 4 °C. Absorbance values were measured at 260 nm using an ultraviolet spectrophotometer:

$$Virus\;concentrat\;ion=\frac{A_{260}\times (dilution\;ratio)}{E_{1\;cm}^{0.1\;\%,260\;nm}}\;$$

#### 3.7.2. Curative Effect of Compounds against TMV *in Vivo*

Growing leaves on *Nicotiana tabacum*, L of the same ages were selected. TMV (concentration of 6 × 10^−3^ mg/mL) was dipped to inoculate the whole leaves. Then the leaves were washed with water and dried. The compound solution was smeared on the left side and the solvent was smeared on the right side for control. The local lesion numbers were then recorded 3–4 days after inoculation [37]. For each compound, three repetitions were conducted. The inhibition rate of the compound was then calculated according to the following formula (‘av’ means average):

$$Inhibition\;rate\;(\%)=\frac{\left[\begin{array}{c} av.local\;lesion\;numbers\;of\;control\\ \left(not\;treated\;with\;compound\right) \end{array}\right]\;-\;(av.local\;lesion\;numbers\;smeared\;with\;drugs)}{av.local\;lesion\;numbers\;of\;control\;(not\;treated\;with\;compound)}\times 100$$

## 4. Conclusions

Polyethyleneglycol sulphonic acid (PEG–OSO_3_H) has been successfully applied as an efficient, environmentally benign polymeric catalyst for the synthesis of a series of 14-aryl-14*H*-dibenzo[*a,j*]xanthenes through one-pot condensation reaction of 2-naphthol with various aromatic aldehydes under solvent-free conditions at 60 °C. This synthetic protocol offers several advantages, including short reaction times, solvent-free conditions, recyclability of the catalyst and high isolated yields of the products. The present investigations concluded that our synthesized xanthenes may have potential as novel chemical compounds with biologically active nitrogenous groups for the development of new anti-viral agents for controlling plant disease caused by TMV and/or other viruses. Results in our work, especially the synthesis of xanthene compounds with an eco-friendly catalyst may help to improve the method of application and possible utilization of this group of compounds in the control of plant virus diseases. Furthermore, extensive investigation in terms of the specific molecular mechanism responsible for anti-viral property of the title compounds is ongoing. 

## Figures and Tables

**Table 1:** Synthesis of xanthenes catalyzed by PEG-OSO_3_H at 60–65 °C from 2-naphthols, aldehydes and cyclic 1,3-dicarbonyl compounds.

Entry	Product	R	Time (min)	Yield (%) ^a^	Melting Point (°C)	
					Found	Literature [Ref.]
1	**3a**	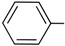	20	96	181–182	182–183 [30]
2	**3b**	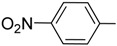	15	92	310–312	311–313 [30]
3	**3c**	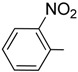	22	96	290–291	292–293 [30]
4	**3d**	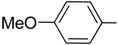	18	91	203–205	204–205 [30]
5	**3e**	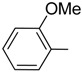	24	95	163–164	163–165 [31]
6	**3f**	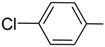	21	90	286–287	287–288 [30]
7	**3g**	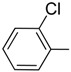	25	93	173–174	172–173 [30]
8	**3h**	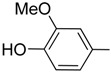	30	85	206–207	208–210 [32]
9	**3i**	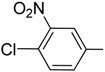	20	97	178–179	-
10	**5**	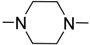	25	90	183–184	-
11	**6**	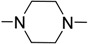	30	94	185–187	-
12	**9**	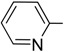	32	92	179–181	-

^a^ Isolated yield.

**Table 2:** Recyclability of the PEG–OSO_3_H for the one-pot condensation reaction of 2-naphthol with benzaldehyde under neat condition.

Entry	Recovery	Time/min	Yields ^a^ (%)
1	0	20	96
2	1	20	95
3	2	20	93
4	3	20	92

^a^ Isolated yields for xanthene (**3a**) after each cycle.
